# Targeted use of heparin, heparinoids, or low-molecular-weight heparin to improve outcome after acute ischaemic stroke: an individual patient data meta-analysis of randomised controlled trials

**DOI:** 10.1016/S1474-4422(13)70079-6

**Published:** 2013-06

**Authors:** William N Whiteley, Harold P Adams, Philip MW Bath, Eivind Berge, Per Morten Sandset, Martin Dennis, Gordon D Murray, Ka-Sing Lawrence Wong, Peter AG Sandercock

**Affiliations:** a Division of Clinical Neurosciences, University of Edinburgh, Bramwell Dott Building, Western General Hospital, Edinburgh, UK; b Department of Neurology, University of Iowa, Iowa City, USA; c Division of Stroke, University of Nottingham, Clinical Sciences Building, City Hospital Campus, Nottingham, UK; d Oslo University Hospital, Department of Haematology, and University of Oslo, Institute of Clinical Medicine, Oslo, Norway; e Centre for Population Health Sciences, University of Edinburgh, Edinburgh, UK; f Division of Neurology, Chinese University of Hong Kong, Prince of Wales Hospital, Shatin, New Territories, Hong Kong, China

## Abstract

**Background:**

Many international guidelines on the prevention of venous thromboembolism recommend targeting heparin treatment at patients with stroke who have a high risk of venous thrombotic events or a low risk of haemorrhagic events. We sought to identify reliable methods to target anticoagulant treatment and so improve the chance of avoiding death or dependence after stroke.

**Methods:**

We obtained individual patient data from the five largest randomised controlled trials in acute ischaemic stroke that compared heparins (unfractionated heparin, heparinoids, or low-molecular-weight heparin) with aspirin or placebo. We developed and evaluated statistical models for the prediction of thrombotic events (myocardial infarction, stroke, deep vein thrombosis, or pulmonary embolism) and haemorrhagic events (symptomatic intracranial or significant extracranial) in the first 14 days after stroke. We calculated the absolute risk difference for the outcome “dead or dependent” in patients grouped by quartiles of predicted risk of thrombotic and haemorrhagic events with random effect meta-analysis.

**Findings:**

Patients with ischaemic stroke who were of advanced age, had increased neurological impairment, or had atrial fibrillation had a high risk of both thrombotic and haemorrhagic events after stroke. Additionally, patients with CT-visible evidence of recent cerebral ischaemia were at increased risk of thrombotic events. In evaluation datasets, the area under a receiver operating curve for prediction models for thrombotic events was 0·63 (95% CI 0·59–0·67) and for haemorrhagic events was 0·60 (0·55–0·64). We found no evidence that the net benefit from heparins increased with either increasing risk of thrombotic events or decreasing risk of haemorrhagic events.

**Interpretation:**

There was no evidence that patients with ischaemic stroke who were at higher risk of thrombotic events or lower risk of haemorrhagic events benefited from heparins. We were therefore unable to define a targeted approach to select the patients who would benefit from treatment with early anticoagulant therapy. We recommend that guidelines for routine or selective use of heparin in stroke should be revised.

**Funding:**

MRC.

## Introduction

Every year, ischaemic stroke kills 2·9 million people and leads to 3·4 million years lived with disability worldwide.^1^, ^2^ Short-term stroke recovery is often complicated by venous thromboembolism (1–5%) and recurrent stroke (1–20%).^3^, ^4^ Prevention of the arterial and venous thromboembolic complications of stroke could contribute to reduction of the burden of stroke-related disabilities.

Although heparins (unfractionated heparin, low-molecular-weight heparin, and heparinoids) can reduce the risk of recurrent ischaemic stroke, deep vein thrombosis, and pulmonary embolism, they also increase the risk of symptomatic intracranial and extracranial haemorrhage.^5^ In stroke, the benefits are exactly offset by the harms, and hence in systematic reviews of grouped data from randomised controlled trials of subcutaneous heparins, there was no net observable effect of anticoagulants on death or disability measured several months after stroke (even in selected subtypes).^6^, ^7^, ^8^

However, some clinicians still use heparin or low-molecular-weight heparin to prevent early recurrent stroke in patients who are felt to be at particularly high risk.^9^, ^10^, ^11^, ^12^, ^13^ In the third International Stroke Trial (IST-3),^14^ 24% of patients were treated with low-dose heparin and 8% were fully anticoagulated with high-dose heparin or warfarin in the first week after stroke, consistent with recent registry data from the USA, Germany, France, and Australia.^9^, ^14^, ^15^, ^16^, ^17^, ^18^ Additionally, regimens of low-dose unfractionated heparin and low-molecular-weight heparin are commonly prescribed for the prevention of deep vein thrombosis and pulmonary embolism in patients with stroke.^19^ National UK and other country guidelines recommend prophylactic doses of heparins for patients with stroke who are deemed at high risk of venous thromboembolism^20^ or those at low risk of bleeding,^21^ although this stratified approach had not previously been studied.

We aimed to test the hypothesis that a policy of using clinical data to target heparins in patients with ischaemic stroke who have a high risk of venous or arterial thromboembolism, and avoiding heparins in patients with a high risk of bleeding, leads to overall better outcomes. Such a hypothesis might also be of relevance to the use of thromboprophylaxis in other groups of medical patients. We therefore undertook a meta-analysis of individual patient data from the five largest randomised trials of unfractionated heparin, heparinoids, and low-molecular-weight heparin in acute ischaemic stroke.^22^

## Methods

### Procedures

We obtained individual patient data from the five largest randomised controlled trials of heparins versus either aspirin or placebo in acute ischaemic stroke that measured post-stroke dependence: IST;^23^, ^24^ the Trial of ORG 10172 in Acute Stroke Treatment (TOAST);^25^ the Tinzaparin in Acute Ischaemic Stroke Trial (TAIST);^26^ the Heparin in Acute Embolic Stroke Trial (HAEST);^27^ and Fraxiparin in Stroke Study for the treatment of ischemic stroke (FISS-tris).^28^ We included only those patients from IST for whom the baseline diagnosis was probable or definite ischaemic stroke. We identified the trials by use of the latest Cochrane review of anticoagulants in acute stroke.^6^ We did not obtain individual patient data from 22 other trials of heparins because they were small (fewer than 100 patients), they were not clearly randomised, or their data were not readily available.

We obtained the following baseline variables from each trial (using the trial definitions): age, sex, delay from stroke onset to randomisation, level of consciousness, facial weakness, arm weakness, leg weakness, presence of atrial fibrillation at randomisation, systolic blood pressure at randomisation, and history of myocardial infarction, stroke, or diabetes mellitus. We used the National Institutes of Health stroke scale (NIHSS) as a measure of initial neurological impairment due to stroke. Where the Scandinavian stroke scale (SSS) was measured, we converted it to the NIHSS with a previously developed conversion algorithm.^29^ IST recorded neither the NIHSS nor the SSS, so we used an algorithm developed in the IST-3 dataset to convert data for eight simple questions of neurological deficit into an NIHSS score.^14^ Three trials recorded the Oxfordshire Community Stroke Project (OCSP) classification at baseline.^30^ For the remaining two trials we converted symptoms recorded in components of the NIHSS scale into an OCSP classification with a previously developed algorithm.^31^

We defined two early outcome events: a composite of thrombotic events within 14 days (any fatal or non-fatal pulmonary embolism, deep vein thrombosis, myocardial infarction, or recurrent ischaemic stroke [not stroke extension alone] as defined by each trial, up to and including 14 days after randomisation); and a composite of haemorrhagic events within 14 days (any recorded fatal or non-fatal intracranial haemorrhage, or extracranial haemorrhages that led to death, transfusion, or surgery, up to and including 14 days post randomisation). We defined the state of being dead or dependent at final follow-up as: in FISS-tris and TAIST, a modified Rankin scale score of 3–6; in IST and HAEST, being either dead or responding yes to the question “did you need help from another person to perform everyday activities within the last 2 weeks?”; or, in TOAST, a Glasgow outcome scale of 1–3 (dead to severely dependent). Some studies measured death or dependence at 3 months and some at 6 months (table 1). We made no specific allowance for differing follow-up times because we made within-study comparisons of the proportion of patients dead or dependent at the end of follow-up.

**Table 1:** Characteristics of included trials

	**IST**	**TOAST**	**FISS-tris**	**HAEST**	**TAIST**
Agent and dose	UFH, 12 500 IU subcutaneously or 5000 IU subcutaneously twice a day	Heparinoid (danaparoid), intravenously adjusted to factor Xa activity	LMWH (nadroparin calcium), 3800 anti-factor Xa IU subcutaneously twice a day	LMWH (dalteparin), 100 IU/kg subcutaneously twice a day	LMWH (tinzaparin), 175 anti-Xa IU/kg or 100 anti-Xa IU/kg subcutaneous daily
Length of treatment	14 days	7 days	10 days	14 days	10 days
Control	Aspirin 300mg or avoid aspirin	Placebo	Aspirin 160 mg	Aspirin 160 mg	Aspirin 300 mg
Randomised	2×2 factorial	1:1	1:1	1:1	1:1:1
Definition of death or dependence	IST scale	Glasgow outcome scale	Modified Rankin scale	IST scale	Modified Rankin scale
Time of follow-up for death or dependence	6 months	3 months	6 months	3 months	6 months

IST=International Stroke Trial. TOAST=Trial of ORG 10172 in Acute Stroke Treatment. FISS-tris=Fraxiparin in Stroke Study for the treatment of ischemic stroke. HAEST=Heparin in Acute Embolic Stroke Trial. TAIST= Tinzaparin in Acute Ischaemic Stroke Trial. IU=international units. LMWH=low-molecular-weight heparin. UFH=unfractionated heparin.

### Predictive models

We searched the medical literature systematically for validated predictive models of short-term haemorrhagic or all thrombotic events after a stroke of moderate to major severity, but found none. We therefore developed and validated predictive models for “all haemorrhagic events” and “all thrombotic events” with the available data.

We first measured the associations between baseline clinical variables and either all thrombotic or all haemorrhagic events in each trial with univariate logistic regression. We calculated a summary estimate of these odds ratios and their 95% CI with random effects meta-analysis, and calculated an *I*^2^ statistic as a measure of heterogeneity between studies. We developed predictive models in the large IST dataset with variables that were plausibly and significantly associated with thrombotic or haemorrhagic events.

As the best test of the external validity of prognostic models is to test them in different datasets, we used the TAIST, TOAST, FISS-tris, and HAEST trials (the test dataset) for evaluation rather than a random split of the data.^32^ We measured discrimination in the test dataset by calculating the area under a receiver operating characteristic curve (AUROCC) and its 95% CI. One interpretation of the AUROCC is the proportion of randomly selected pairs of patients with and without an event, in which the patient with an event has a higher predicted risk of an event than the patient without an event. An AUROCC of 0·5 indicates no better discrimination than chance, and an AUROCC of 1 indicates perfect discrimination. We measured calibration by plotting the predicted versus observed risks of events per quintile of predicted risk, and calculating the Hosmer Lemeshow χ^2^ statistic, where 2P>0·05 is one measure of a well calibrated model.

To measure the effect of missing baseline variables on the strength of associations in the final multivariate model, we repeated the analysis with ten imputations of missing variables, using complete baseline variables as predictors of missing values with logistic regression equations.^33^ We also developed a model containing those available variables that were recommended in recent UK National Institute of Health and Care Excellence (NICE) guidelines^21^ to predict venous thromboembolism in patients admitted to hospital: age over 60 years, presence of leg weakness, and presence of comorbidities.

### Statistical analysis

To establish whether the predicted risk of thrombotic or haemorrhagic events was associated with the response to heparins (measured by the proportion of patients dead or dependent at final follow-up), we divided the population of each of the five trials into sixteen groups, defined by quartiles of predicted risk of thrombotic events and quartiles of predicted risk of haemorrhagic events. For each group in each trial, we calculated the excess risk of death or disability in patients allocated to heparins compared with patients allocated to aspirin or placebo in each trial (intention to treat), and then used random effects meta-analyses to pool the risk differences from each trial in a summary estimate. This ensures that treated and untreated patients are compared within and not between trials, which maintains the balance achieved by randomisation as far as is possible. We repeated the analysis comparing low-dose heparins to placebo or aspirin, and high-dose heparins to placebo or aspirin. Because dichotomous outcomes are not as sensitive to small effects as a more statistically efficient ordinal approach, we also analysed disability and death on a common four-point disability scale with ordinal logistic regression.^34^ We tested the statistical significance of a multiplicative interaction between [(predicted risk of thrombosis) – (predicted risk of haemorrhage)] and treatment with heparins. We used Stata 11 for the analysis.

### Role of the funding source

The sponsors of the study had no role in study design, data collection, data analysis, data interpretation, or writing of the report. The corresponding author had full access to all the data in the study and had final responsibility for the decision to submit for publication.

## Results

We obtained data for 22 655 patients with ischaemic stroke who were randomised to either an anticoagulant regimen (unfractionated heparin, low-molecular-weight heparin, or heparinoid) or to aspirin or placebo from the IST, TOAST, FISS-tris, HAEST, and TAIST studies. The key design features of each trial are summarised in table 1. The clinical features of patients at baseline in each study are summarised in the appendix. By 14 days after randomisation, about 1 in 20 patients had had a thrombotic event (1302 [5·7%]; table 2), most of which were recurrent ischaemic strokes (817 [3·6%]). Significant haemorrhagic events were recorded about a third as often as thrombotic events by 14 days after randomisation (374 [1·7%]; table 2), with similar numbers of extracranial and intracranial haemorrhages (207 [0·9%] and 184 [0·8%], respectively). About two-thirds of patients were dead or dependent at the time of last follow-up (13 230 [58·4%]), although the proportion was lower in the TOAST and FISS-tris trials (table 2). Patients randomly assigned to a heparin regimen had a 1·6% absolute increase in the risk of haemorrhagic events and a 1·4% reduction in the risk of thrombotic events compared with those assigned to aspirin or placebo. For low-dose heparin versus aspirin or placebo, the proportions were a 0·5% increase and a 1·4% decrease. Patients who had a haemorrhagic or thrombotic event within 2 weeks of stroke had a high risk of death or dependence: 82% of patients who had a haemorrhagic event and 88% of patients who had a thrombotic event were dead or dependent at final follow-up.

**Table 2:** Thrombotic and haemorrhagic events within 14 days and death or dependence at final follow-up

	**IST (N=18836)**	**TOAST (N=1281)**	**FISS-tris N=603)**	**HAEST (N=449)**	**TAIST (N=1486)**	**Total (N=22655)**
Deep venous thrombosis	21 (0·1%)	12 (0·9%)	3 (0·5%)	5 (1·1%)	16 (1·1%)	57 (0·3%)
Pulmonary embolism	126 (0·7%)	6 (0·5%)	0 (0·0%)	1 (0·2%)	11 (0·7%)	144 (0·6%)
Ischaemic stroke	646* (3·4%)	62 (4·8%)	31 (5·1%)	36 (8·0%)	42 (2·8%)	817 (3·6%)
Myocardial infarction	362 (1·9%)	17 (1·3%)	..	4 (0·9%)	..	383 (1·7%)
Any thrombotic events	1141 (6·1%)	36 (2·8%)	16 (2·7%)	45 (10·0%)	64 (4·3%)	1302 (5·7%)
Significant intracranial haemorrhage	127 (0·7%)	21 (1·6%)	15 (2·5%)	10 (2·2%)	11 (0·7%)	184 (0·8%)
Major extracranial haemorrhage	163 (0·9%)	31 (2·4%)	2 (0·3%)	3 (0·7%)	8 (0·5%)	207 (0·9%)
Any haemorrhagic event	285 (1·5%)	43 (3·4%)	14 (2·3%)	13 (2·9%)	19 (1·3%)	374 (1·7%)
Dead or dependent†	11 654 (61·9%)	294 (23·0%)	157 (26·0%)	294 (65·5%)	831 (55·9%)	13 230 (58·4%)

Data are n (%).

* Includes patients where recurrent stroke subtype was uncertain.

† 143 (<1%) of patients had missing dead or dependent status at final follow-up. Each number signifies an individual with a haemorrhagic or a thrombotic event. 27 individuals had both a haemorrhagic and a thrombotic event. Events are uniformly counted from 14 days after randomisation, hence minor differences from trial publication in number of events.

**Figure 1:**
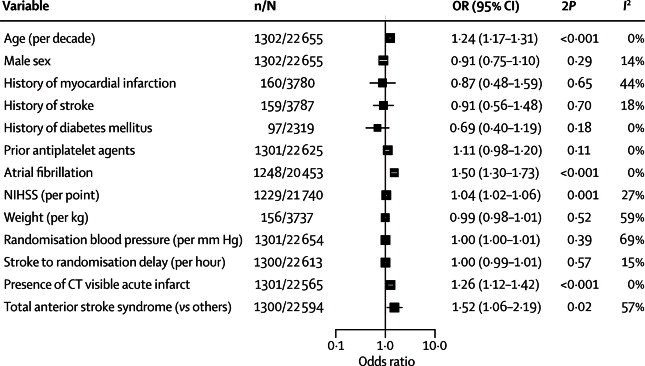
Association of baseline variables with thrombotic events—myocardial infarction, recurrent ischaemic stroke, deep vein thrombosis, and pulmonary embolism Each square represents the point estimate from a random effects meta-analysis across trials, and the horizontal line the 95% CI. N=number of patients. n=number of events in each meta-analysis. NIHSS=National Institutes of Health stroke scale.

In univariate analysis, four factors were significantly and consistently associated across trials with the risk of thrombotic events after stroke: increasing age, presence of atrial fibrillation, a CT-visible infarction, and an increasing NIHSS score (figure 1). A prediction model constructed with these four variables (table 3) using data from the IST dataset discriminated moderately well between those patients who did have a thrombotic event by 14 days post-stroke and those who did not, both in the development dataset (AUROCC 0·60, 95% CI 0·58–0·62) and in the test dataset (0·63, 0·59–0·67). There was no improvement in model fit with non-linear transformations of the NIHSS score, age, delay from stroke, or systolic blood pressure. Multiple imputation of missing variables made little difference to the magnitude or directions of the association seen in the model created in the development dataset. In the test dataset, there was a positive gradient of thrombotic events by quintiles of predicted risk of thrombotic events (table 4), from a 2·0% risk in the lowest quintile to 7·0% in the highest quintile.

**Table 3:** Prediction models for thrombotic and haemorrhagic events developed in the IST dataset

	**Odds ratio (95% CI)**	**p**
**Prediction model for all thrombotic events**		
Age (per year)	1·02 (1·02–1·03)	<0·0001
Presence of atrial fibrillation	1·24 (1·02–1·51)	0·007
Presence of CT evidence of recent cerebral ischaemia	1·18 (1·03–1·35)	0·014
NIHSS (per point)	1·04 (1·02–1·05)	<0·0001
**Prediction model for all haemorrhagic events**		
Age (per year)	1·01 (1·00–1·02)	0·04
Presence of atrial fibrillation	1·09 (0·78–1·50)	0·60
NIHSS (per point)	1·06 (1·04–1·09)	<0·0001

NIHSS=National Institutes of Health stroke scale.

**Table 4:** Number of thrombotic and haemorrhagic events by quintiles of predicted risk of event in evaluation datasets (TAIST, TOAST, FISS-tris, HAEST)

	**N**	**Number of events (%)**
Quintile of predicted risk of thrombotic event*		
1	749	15 (2·0%)
2	744	23 (3·1%)
3	745	29 (3·9%)
4	746	41 (5·5%)
5	745	52 (7·0%)
Total	3729	160 (4·3%)
**Quintile of predicted risk of haemorrhagic event**†		
1	763	12 (1·6%)
2	763	13 (1·7%)
3	764	18 (2·4%)
4	762	11 (1·4%)
5	763	35 (4·6%)
Total	3815	89 (2·3%)

N=number of patients.

* Hosmer Lemeshow χ^2^=3·2, p=0·35.

† Hosmer Lemeshow χ^2^=4·2, p=0·23.

Models to predict symptomatic venous thromboembolism alone discriminated moderately well between patients who developed venous thromboembolism and those who did not in the test dataset when based on variables that predicted all thrombotic events (AUROCC 0·66, 95% CI 0·59–0·73), but not when based on available variables suggested in the NICE guidelines: age older than 60 years, presence of leg weakness, and presence of comorbidities (0·49, 0·46–0·52).

**Figure 2:**
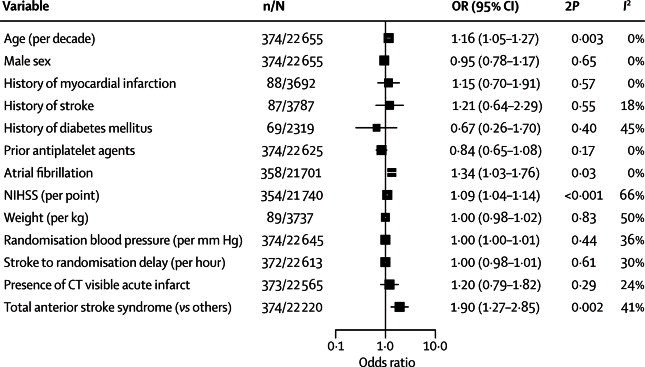
Association of baseline variables with haemorrhagic events (intracranial or extracranial haemorrhage) Each square represents the point estimate from a random effects meta-analysis across trials, and the horizontal line the 95% CI. N=number of patients, n=number of events in each meta-analysis.

Increasing age, increasing NIHSS score, and the presence of atrial fibrillation were associated with an increased risk of any haemorrhagic event (figure 2). A prediction model constructed from these three variables (table 3) discriminated only moderately between those patients who developed a haemorrhagic event within 14 days in the IST development dataset (AUROCC 0·61, 95% CI 0·58–0·64) with a similar performance in the test dataset (0·60, 0·55–0·64). In the test dataset, there was a moderate gradient of increased absolute risk of haemorrhagic events by quintiles of predicted risk of haemorrhage from the lowest (1·6%) to the highest (4·6%) quintile (table 4).

Patients in each trial were divided into sixteen groups, defined by quartiles of risk of all haemorrhagic events and quartiles of risks of all thrombotic events (table 5). We found that no group had a statistically significant benefit of heparins over aspirin or placebo for the prevention of death or disability at the time of last follow-up. In none of the 16 groups was there evidence of significant heterogeneity between the risk differences from the different trials. There was no visible pattern or trend of increasing benefit or harm across the groups.

**Table 5:** Absolute risk difference of death or disability at final follow-up between patients treated with heparins and those treated with aspirin or placebo across all five trials, presented by quartiles of risk of haemorrhagic events and quartiles of risk of thrombotic events (absolute risk difference for whole population 0·00 [–0·01 to 0·01])

		**Quartiles of predicted risk of haemorrhagic events**			
		1 (lowest)	2	3	4 (highest)
Quartiles of predicted risk of thrombotic events*					
	1 (low)	−0·02 (−0·05 to 0·02); N=3190	−0·02 (−0·08 to 0·04); N=1140	−0·16 (−0·34 to 0·02); N=129	..; N=0
	2	−0·02 (−0·07 to 0·04); N=1074	0·01 (−0·04 to 0·05); N=2674	0·03 (−0·03 to 0·08); N=1336	−0·08 (−0·27 to 0·10); N=98
	3	0·00 (−0·17 to 0·16); N=151	−0·04 (−0·09 to 0·02); N=1168	0·03 (−0·03 to 0·09); N=2783	0·01 (−0·04 to 0·06); N=1074
	4 (high)	..; N=3	−0·02 (−0·18 to 0·15); N=121	0·01 (−0·04 to 0·06); N=1036	0·00 (−0·02 to 0·02); N=4025

* Data are absolute % increase in risk of death or disability with heparins (95% CI); total number of patients. Each risk difference was calculated with a random effects meta-analysis across trials. Negative numbers indicate fewer patients dead or dependent after treatment with heparin. N indicates the total number of patients across trials in each cell. In no group was p<0·05 for risk difference; in no group was between-trial p_heterogeneity_<0·2.

The results were similar when examining patients from IST alone; when examining patients from the HAEST, TAIST, TOAST, and FISS-tris trials together; when comparing patients randomly assigned to low-dose heparins with those randomised to aspirin or placebo; when comparing patients randomised to high-dose heparins with those randomised to aspirin or placebo; or where death at final follow-up was the outcome of interest. The term [(predicted thrombosis–predicted risk of haemorrhage) × heparin] was not significant in an ordinal logistic regression model (p=0·43).

## Discussion

We have not been able to define a strategy that can reliably select the patients with ischaemic stroke who are most likely to benefit and least likely to be harmed by early heparin therapy. This stratified analysis of individual patient data from the five largest trials of heparins in acute stroke therefore provides no support for targeting the use of heparin, heparinoids, or low-molecular-weight heparin after stroke for the prevention of thrombotic events to reduce post-stroke death or disability using risk models based on simple clinical variables (including those proposed by NICE).

Higher age, greater stroke severity, and the presence of atrial fibrillation were associated with an increased risk of both thrombotic and haemorrhagic events. Therefore, patients at a higher predicted risk of thrombotic events were also at a higher risk of haemorrhagic events, and no single variable discriminated reliably between the risk of thrombosis and haemorrhage.

This study has several limitations. The models that we produced were only moderately predictive of recurrent thrombotic or haemorrhagic events. Many prognostic models developed in patients with stroke have only poor to moderate predictive performance: for example, prediction models of bleeding (AUROCC 0·67),^35^ recurrent stroke (0·61–0·68),^36^ and deep vein thrombosis (0·57).^22^ The accuracy of the models developed in our analysis was similar. However, unlike observational cohorts, the assessment of the effect of heparins was unbiased because they were randomly allocated. Our predictive models might have been improved if we had been able to test important and plausible predictive variables that were missing in this dataset, such as renal failure, history of a previous venous thrombosis, or gastrointestinal haemorrhage. However, these events are fairly infrequent in clinical stroke practice. Because most thrombotic and haemorrhagic events occur in people without these risk factors, the addition of these variables is unlikely to have materially improved the performance and accuracy of predictive models.^22^

Variables were defined and obtained in different ways in different trials, which could have added random error to each of the variables (particularly measures of stroke severity). We used data from large randomised trials in which the primary focus was the collection of data for death or dependence at end of follow-up, rather than the collection of data on recurrent events or venous thromboembolism. Some important events were probably missed, which might have limited the discriminative performance of the predictive models. Despite this, the proportion of patients with symptomatic events was within the bounds of those recorded by contemporaneous studies where the primary focus was the proportion of patients with short term post stroke complications.^37^ We identified trials found in a recent Cochrane systematic review,^6^ rather than updating the search; however, we believe that we are unlikely to have missed any large trials of heparins in acute stroke.

This analysis provides no support for the guideline recommendations and common clinical practice of individualised risk assessment of haemorrhagic or thrombotic events in patients with ischaemic stroke when making a decision whether to prescribe a heparin. To support such a practice, the analysis would need to show that prediction of early risk could be validated against the observed risk of thrombotic and haemorrhagic events, and that those patients who were at high predicted risk of further thrombotic events and low risk of haemorrhagic events were less likely to be dead or dependent after treatment with a heparin. Because we have analysed most of the available randomised evidence for patients with stroke, further large-scale observational data would be necessary to develop better predictive models, and more randomised trials of heparins would be needed to test whether the use of these models improved the effect of a heparin on death and disability. Most of the prediction power of statistical models to predict early recurrent thrombotic and haemorrhagic events would still probably be determined by age and severity of symptoms, so additional factors (not correlated with age or symptom severity) would need to be identified. Candidate variables for the prediction of haemorrhage with heparin include brain microbleeds, renal impairment, a history of haemorrhagic events, or more detailed analysis of brain imaging findings; for the prediction of thrombotic events, variables include a history of venous thromboembolism, an inherited thrombophilia, or cancer.

Guidelines from the American College of Physicians^38^ and NICE^21^ recommend an individualised assessment of risk of venous thromboembolism and the risk of haemorrhage before initiating a heparin in patients with stroke. Several potential individual risk predictors are given in both guidelines, although the discriminative performance of these factors was not stated. Both of these guidelines gave their recommendations by summing the benefit of treatment in terms of number of thrombotic events and the harms in terms of numbers of haemorrhagic events. Their analysis assumes that equal weight can be placed on non-fatal thrombotic and haemorrhagic events, which can be challenged because non-fatal pulmonary embolism is usually of less clinical significance than non-fatal intracranial haemorrhage.^5^ By using the primary outcome of the stroke studies—a global measure of dependency—we have not needed to make any assumption as to the weighting to be placed on haemorrhagic or thrombotic events. On our dependency scale, there was no evidence of benefit of heparins after ischaemic stroke in any risk group.

Heparin, heparinoid, or low-molecular-weight heparin treatment did not reduce the risk of death or dependency in patients with ischaemic stroke who have a higher risk of thrombotic events or a lower risk of haemorrhagic events, either because there was no difference, or because our models were only moderately predictive of events. In view of the lack of evidence for heparin prophylaxis in reducing mortality in other categories of high-risk medical patients, and in stroke, these data suggest current guideline recommendations for routine or selective use of heparin in stroke (and perhaps other patients) should be revised.
