# Supplementary material for: Targeted use of heparin, heparinoids, or low-molecular-weight heparin to improve outcome after acute ischaemic stroke: an individual patient data meta-analysis of randomised controlled trials
Source: Lancet Neurol. 2013 Jun;12(6):539–45. doi: 10.1016/S1474-4422(13)70079-6 (PMC3671273; doi:10.1016/S1474-4422(13)70079-6)
Supplement: Supplementary appendix [file mmc1.pdf]

THE LANCET Neurology

Supplementary webappendix

This webappendix formed part of the original submission and has been peer reviewed. We post it as supplied by the authors.

Supplement to: Whiteley WN, Adams HP Jr, Bath PMW, et al. Targeted use of heparin, heparinoids, or low-molecular-weight heparin to improve outcome after acute ischaemic stroke: an individual patient data meta-analysis of randomised controlled trials. *Lancet Neurol* 2013; published online May 2. http://dx.doi.org/10.1016/S1474-4422(13)70079-6.

Targeted use of heparin, heparinoids, or low-molecularweight heparin to improve outcome after acute ischaemic stroke: an individual patient data meta-analysis of randomised controlled trials

SUPPLEMENTAL MATERIAL

		For the prevention of recurrent stroke		For the prevention of venous thromboembolism			
**Guideline organisation and date**	**Title**	**Uncertain benefit of targeting**	**Avoid all**	**Treat all**	**Avoid all**	**Treat at 'high risk' of VTE**	**Avoid if 'high risk' of haemorrhage**
**Acute stroke guidelines**							
Institute for Clinical Systems Improvement 2010	Diagnosis and treatment of ischemic stroke	✓	✓	✓			
Joint Stroke Guideline Development Committee of the American Academy of Neurology and the American Stroke Association 2002	Anticoagulants and antiplatelet agents in acute ischemic stroke		✓			✓	✓
American College of Chest Physicians 2012	Antithrombotic and thrombolytic therapy for ischemic stroke		✓	✓			
National Institute for Health and Clinical Excellence 2008	Diagnosis and Initial management of acute stroke and transient ischaemic attack		✓			✓	
American Association of Neuroscience Nurses 2008	Guide to the care of the hospitalized patient with ischemic stroke					✓	✓
Canadian best practice recommendations for stroke care 2010	Acute stroke management		✓			✓	✓
Scottish Intercollegiate Guidelines Network 2008	Management of patients with stroke or TIA: assessment, investigation, immediate		✓			✓	

Guideline organisation and date	Title	For the prevention of recurrent stroke		For the prevention of venous thromboembolism			
		**Uncertain benefit of targeting**	**Avoid all**	**Treat all**	**Avoid all**	**Treat at 'high risk' of VTE**	**Avoid if 'high risk' of haemorrhage**
	management and secondary prevention						
Singapore Ministry of Health 2009	Stroke and transient ischaemic attacks. Assessment, investigation, immediate management and secondary prevention		✓		✓		
European Stroke Organisation 2008	Guidelines for the management of Ischaemic Stroke		✓			✓	
American Heart Association/ American Stroke Association Stroke Council, 2007	Guidelines for the Early Management of Adults With Ischemic Stroke		✓				
National Stroke Foundation, Australia 2010	Clinical Guideline for Stroke Management					✓	
**Prevention of VTE guidelines**							
Institute for Clinical Systems Improvement 2011	Venous thromboembolism prophylaxis			✓			
American College of Physicians 2011	Venous thromboembolism prophylaxis in hospitalized patients					✓	✓
National Institute for Health and Clinical Excellence 2010	Venous thromboembolism: reducing the risk					✓	✓
Scottish Intercollegiate Guidelines Network 2010	Prevention and management of venous thromboembolism					✓	
Finnish Medical Society Duodecim 2008	Prevention of venous thromboembolism.			✓			

Supplementary table 1 Baseline characteristics of patients

	IST		TOAST		FISS-tris		HAEST		TAIST		Total	
	No.	%	No.	%	No.	%	No.	%	No.	%	No.	%
	N=18836		N=1281		N=603		N=449		N=1486		N=22655	
**Age (years)**												
<50	1042	5.5%	137	10.7%	53	8.8%	1	0.2%	73	4.9%	1306	5.8%
50-59	1759	9.3%	231	18.0%	95	15.8%	8	1.8%	136	9.2%	2229	9.8%
60-69	4254	22.6%	384	30.0%	182	30.2%	45	10.0%	330	22.2%	5195	22.9%
70-79	6472	34.4%	420	32.8%	217	36.0%	167	37.2%	561	37.8%	7837	34.6%
>=80	5309	28.2%	109	8.5%	56	9.3%	228	50.8%	386	26.0%	6088	26.9%
**Sex**												
Women	8741	46.4%	504	39.3%	245	40.6%	248	55.2%	678	45.6%	10416	46.0%
Men	10095	53.6%	777	60.7%	358	59.4%	201	44.8%	808	54.4%	12239	54.0%
**Time from stroke onset to randomisation (hrs)**												
0-6	2996	15.9%	48	3.7%	9	1.5%	24	5.3%	53	3.6%	3130	13.8%
6-12	3986	21.2%	350	27.3%	38	6.3%	101	22.5%	219	14.7%	4694	20.7%
12-24	5408	28.7%	862	67.3%	205	34.0%	180	40.1%	428	28.8%	7083	31.3%
>24h	6446	34.2%	6	0.5%	350	58.0%	143	31.8%	761	51.2%	7706	34.0%
missing	0	0.0%	15	1.2%	1	0.2%	1	0.2%	25	1.7%	42	0.2%
**History of MI**												
no	-	-	928	72.4%	549	91.0%	292	65.0%	1229	82.7%	2998	13.2%
yes	-	-	343	26.8%	50	8.3%	157	35.0%	232	15.6%	782	3.5%
missing	-	-	10	0.8%	4	0.7%	0	0.0%	25	1.7%	18875	83.3%
**History of stroke**												
no	-	-	1038	81.0%	500	82.9%	341	75.9%	1271	85.5%	3150	13.9%
yes	-	-	232	18.1%	103	17.1%	108	24.1%	194	13.1%	637	2.8%
missing	-	-	11	0.9%	0	0.0%	0	0.0%	21	1.4%	18868	83.3%

	IST		TOAST		FISS-tris		HAEST		TAIST		Total	
	No.	%	No.	%	No.	%	No.	%	No.	%	No.	%
**History of hypertension**												
no	-	-	413	32.2%	169	28.0%	270	60.1%	726	48.9%	1578	7.0%
yes	-	-	850	66.4%	434	72.0%	179	39.9%	728	49.0%	2191	9.7%
missing	-	-	18	1.4%	0	0.0%	0	0.0%	32	2.2%	18886	83.4%
**History of diabetes**												
no	-	-	893	69.7%	356	59.0%	383	85.3%	0	0.0%	1632	7.2%
yes	-	-	374	29.2%	247	41.0%	66	14.7%	0	0.0%	687	3.0%
missing	-	-	14	1.1%	0	0.0%	0	0.0%	1486	100.0%	20336	89.8%
**Antiplatelet drug prior to randomisation**												
no	15007	79.7%	732	57.1%	402	66.7%	248	55.2%	922	62.0%	17311	76.4%
yes	3829	20.3%	528	41.2%	193	32.0%	201	44.8%	563	37.9%	5314	23.5%
missing	0	0.0%	21	1.6%	8	1.3%	0	0.0%	1	0.1%	30	0.1%
**OCSP stroke classification**												
TACS	4452	23.6%	439	34.3%	34	5.6%	102	22.7%	523	35.2%	5550	24.5%
PACS	7627	40.5%	148	11.6%	169	28.0%	238	53.0%	473	31.8%	8655	38.2%
LACS	4524	24.0%	540	42.2%	279	46.3%	71	15.8%	422	28.4%	5836	25.8%
POCS	2177	11.6%	153	11.9%	121	20.1%	36	8.0%	66	4.4%	2553	11.3%
missing	56	0.3%	1	0.1%	0	0.0%	2	0.4%	2	0.1%	61	0.3%
**NIH Stroke Scale[1]**												
0-5	1309	6.9%	449	35.1%	294	48.8%	109	24.3%	153	10.3%	2314	10.2%
6-10	3893	20.7%	463	36.1%	214	35.5%	135	30.1%	481	32.4%	5186	22.9%
11-15	8680	46.1%	207	16.2%	68	11.3%	78	17.4%	455	30.6%	9488	41.9%
16-20	2389	12.7%	75	5.9%	18	3.0%	73	16.3%	226	15.2%	2781	12.3%
>20	1654	8.8%	87	6.8%	5	0.8%	54	12.0%	171	11.5%	1971	8.7%

[1] Converted from Scandinavian stroke (TAIST, HAEST) or markers of stroke severity (IST)

	IST		TOAST		FISS-tris		HAEST		TAIST		Total	
	No.	%	No.	%	No.	%	No.	%	No.	%	No.	%
**Systolic blood pressure (mmHg)**												
<145	6031	32.0%	434	33.9%	227	37.6%	106	23.6%	477	32.1%	7275	32.1%
146-170	7341	39.0%	594	46.4%	239	39.6%	172	38.3%	608	40.9%	8954	39.5%
>170	5464	29.0%	253	19.8%	133	22.1%	171	38.1%	395	26.6%	6416	28.3%
**Atrial fibrillation**												
no	14792	78.5%	1166	91.0%	603	100.0%	31	6.9%	1305	87.8%	17897	79.%
yes	3090	16.4%	115	9.0%	0	0.0%	418	93.1%	181	12.2%	3804	16.8%
missing	954[2]	5.1%	0	0.0%	0	0.0%	0	0.0%	0	0.0%	954	4.2%
**Evidence of infarction baseline CT**												
no	12449	66.1%	952	74.3%	160	26.5%	103	22.9%	587	39.5%	14251	62.9%
yes	6387	33.9%	329	25.7%	354	58.7%	346	77.1%	898	60.4%	8314	36.7%
missing	0	0.0%	0	0.0%	89	14.8%	0	0.0%	1	0.1%	90	0.4%

Abbreviations. OCSP=Oxfordshire community stroke project; TACS=total anterior circulation syndrome; PACS=partial anterior circulation stroke syndrome; LACS=lacunar stroke syndrome; POCS=posterior circulation stroke syndrome.

[2] Atrial fibrillation not recorded in the pilot phase of the trial (n=954).

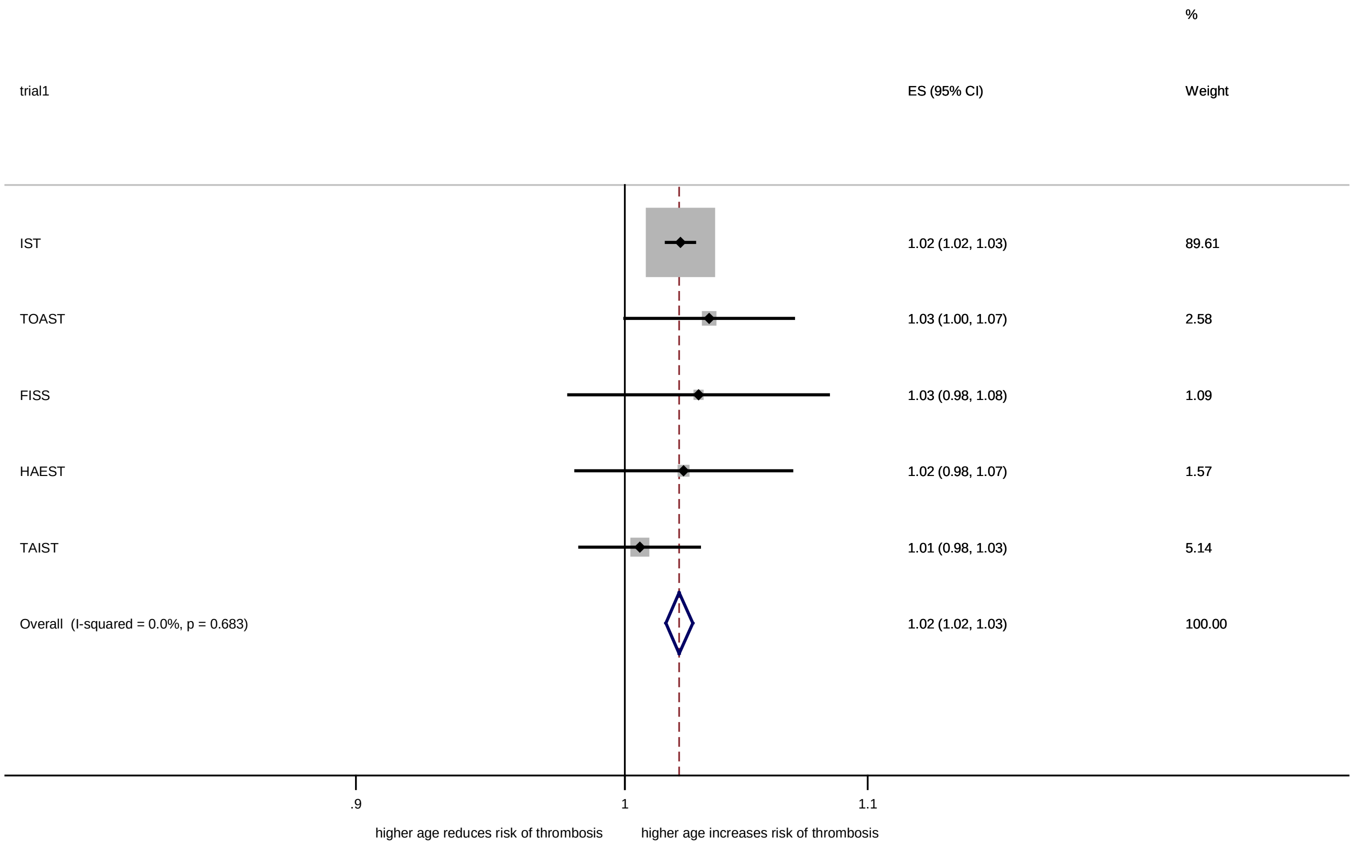

Supplementary Figure 1. The odds ratio (ES) for the association between a one year increase in age with thrombotic events 2 weeks post ischaemic stroke. IST: International Stroke Trial; TOAST: Trial of ORG 10172 in Acute Stroke Treatment; TAIST: Tinzaparin in Acute Ischaemic Stroke Trial; HAEST: Heparin in Acute Embolic Stroke Trial; FISS-tris: Fraxiparin in Stroke Study for the treatment of ischemic stroke. I squared: a measure of statistical heterogeneity.

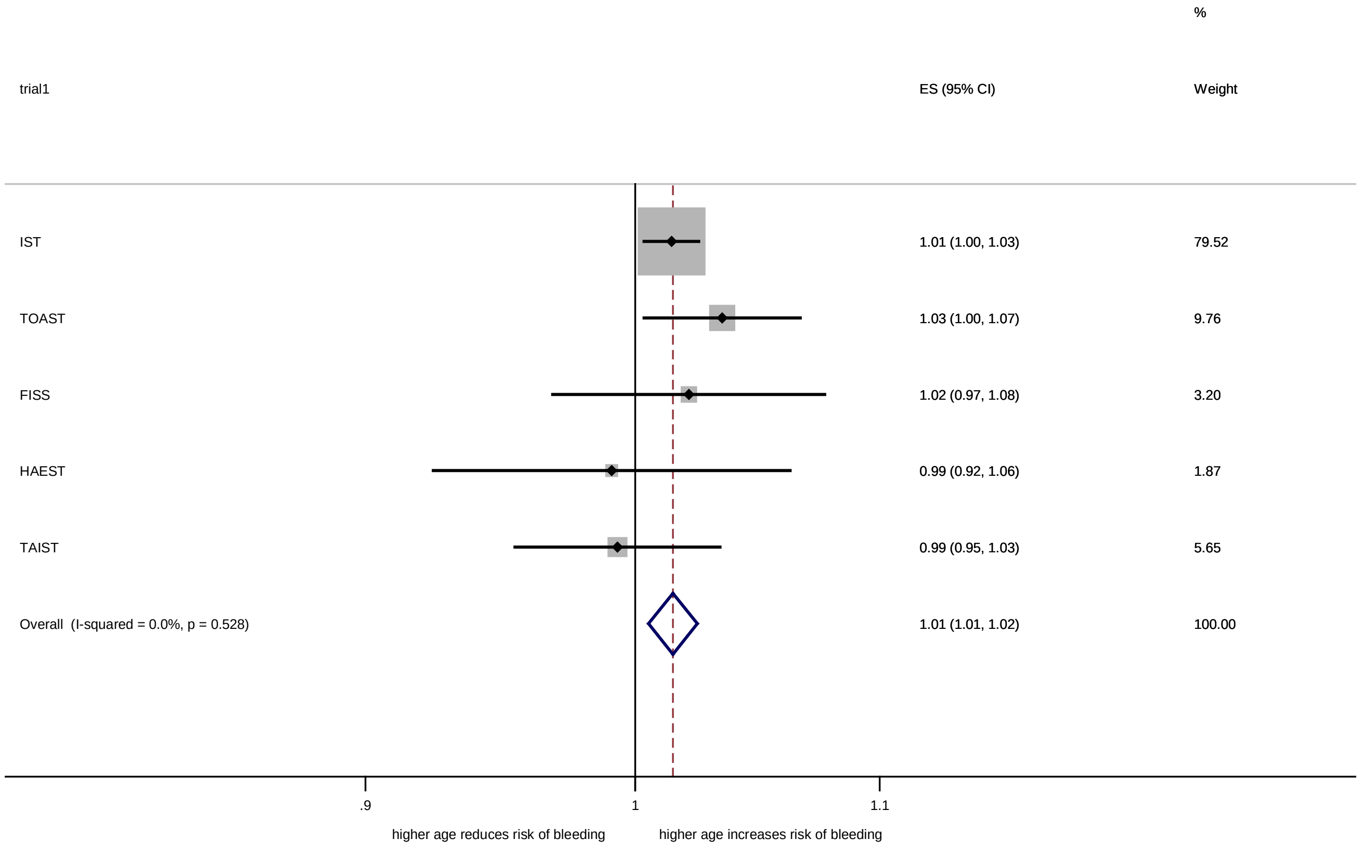

Supplementary Figure 2. The odds ratio (ES) between a one year increase in age with haemorrhagic events 2 weeks post ischaemic stroke IST: International Stroke Trial; TOAST: Trial of ORG 10172 in Acute Stroke Treatment; TAIST: Tinzaparin in Acute Ischaemic Stroke Trial; HAEST: Heparin in Acute Embolic Stroke Trial; FISS-tris: Fraxiparin in Stroke Study for the treatment of ischemic stroke. I squared: a measure of statistical heterogeneity.

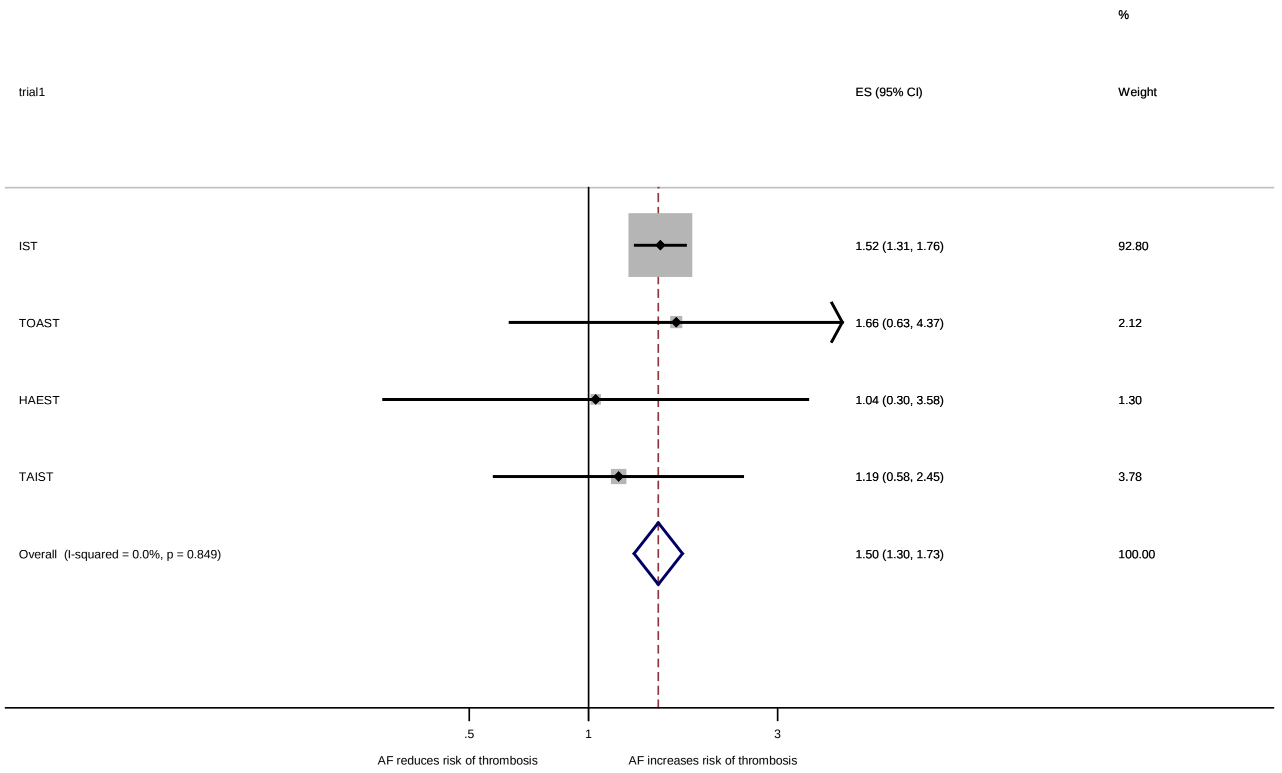

Supplementary Figure 3. The odds ratio (ES) between the presence of AF with thrombotic events 2 weeks post ischaemic stroke. IST: International Stroke Trial; TOAST: Trial of ORG 10172 in Acute Stroke Treatment; TAIST: Tinzaparin in Acute Ischaemic Stroke Trial; HAEST: Heparin in Acute Embolic Stroke Trial; FISS-tris: Fraxiparin in Stroke Study for the treatment of ischemic stroke. I squared: a measure of statistical heterogeneity. AF defined uniformly across trials.

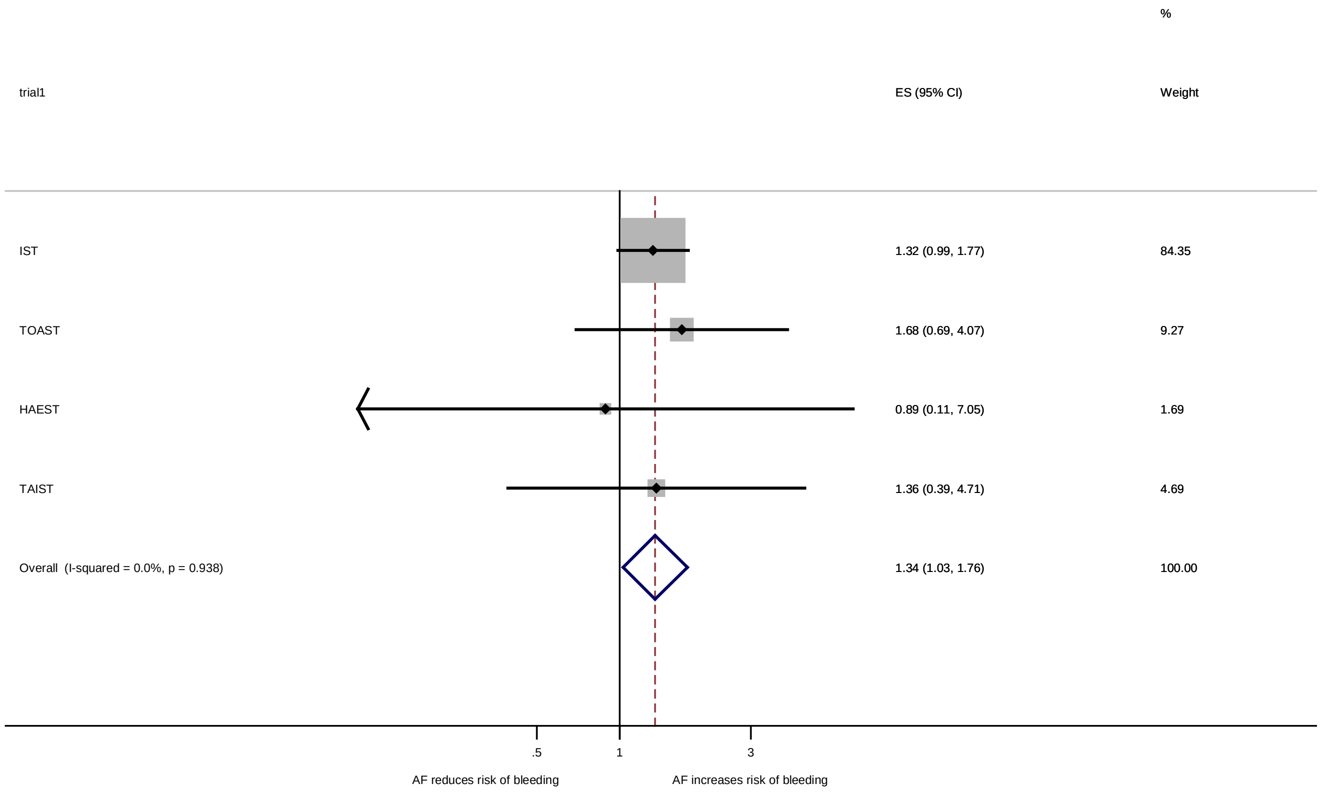

Supplementary Figure 4. The odds ratio (ES) between the presence of AF with haemorrhagic events 2 weeks post ischaemic stroke. IST: International Stroke Trial; TOAST: Trial of ORG 10172 in Acute Stroke Treatment; TAIST: Tinzaparin in Acute Ischaemic Stroke Trial; HAEST: Heparin in Acute Embolic Stroke Trial; FISS-tris: Fraxiparin in Stroke Study for the treatment of ischemic stroke. I squared: a measure of statistical heterogeneity. AF defined uniformly across trials.

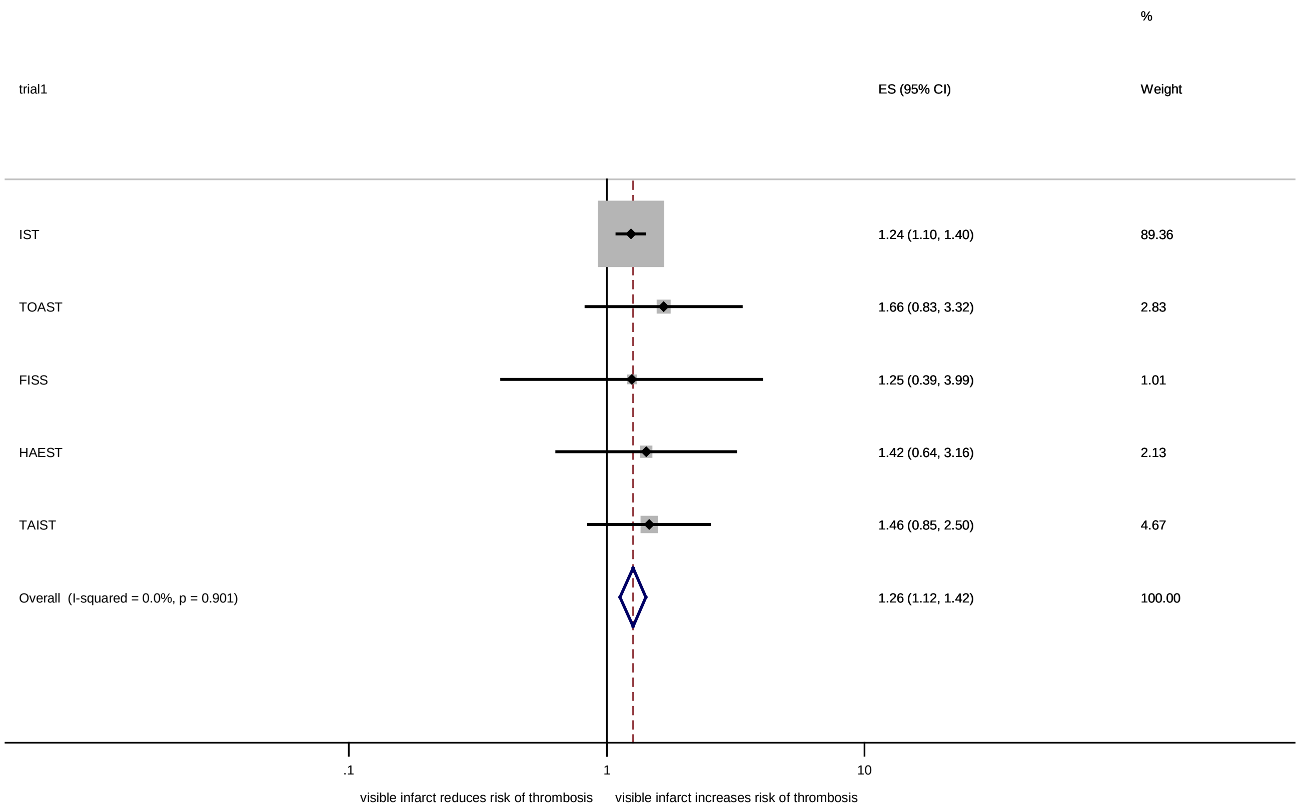

Supplementary Figure 5. The odds ratio (ES) between the presence of CT visible infarct with thrombotic events 2 weeks post ischaemic stroke. IST: International Stroke Trial; TOAST: Trial of ORG 10172 in Acute Stroke Treatment; TAIST: Tinzaparin in Acute Ischaemic Stroke Trial; HAEST: Heparin in Acute Embolic Stroke Trial; FISS-tris: Fraxiparin in Stroke Study for the treatment of ischemic stroke. I squared: a measure of statistical heterogeneity.

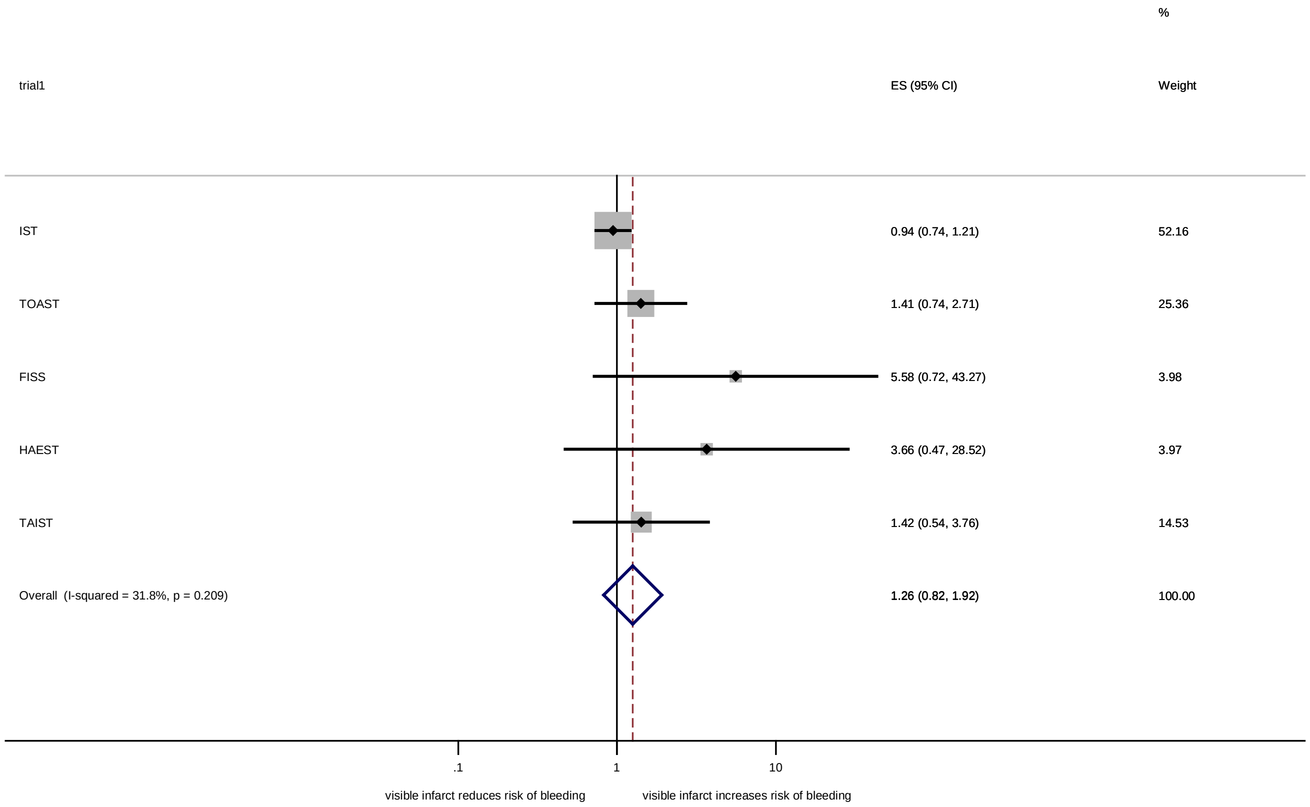

Supplementary Figure 6. The odds ratio (ES) between the presence of CT visible infarct with haemorrhagic events 2 weeks post ischaemic stroke. IST: International Stroke Trial; TOAST: Trial of ORG 10172 in Acute Stroke Treatment; TAIST: Tinzaparin in Acute Ischaemic Stroke Trial; HAEST: Heparin in Acute Embolic Stroke Trial; FISS-tris: Fraxiparin in Stroke Study for the treatment of ischemic stroke. I squared: a measure of statistical heterogeneity.

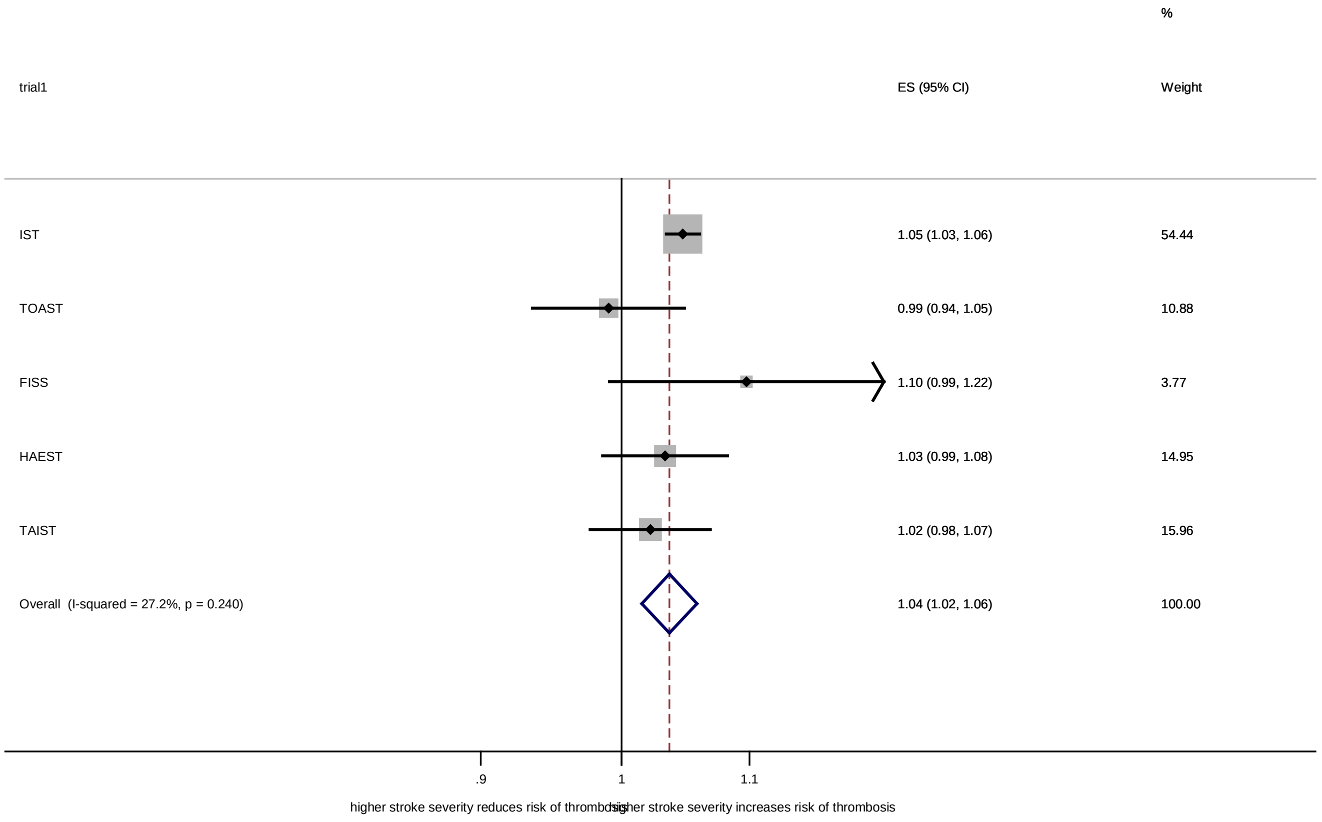

Supplementary figure 7. The odds ratio (ES) between a one year increase in NIHSS with thrombotic events 2 weeks post ischaemic stroke. IST: International Stroke Trial; TOAST: Trial of ORG 10172 in Acute Stroke Treatment; TAIST: Tinzaparin in Acute Ischaemic Stroke Trial; HAEST: Heparin in Acute Embolic Stroke Trial; FISS-tris: Fraxiparin in Stroke Study for the treatment of ischemic stroke. I squared: a measure of statistical heterogeneity.

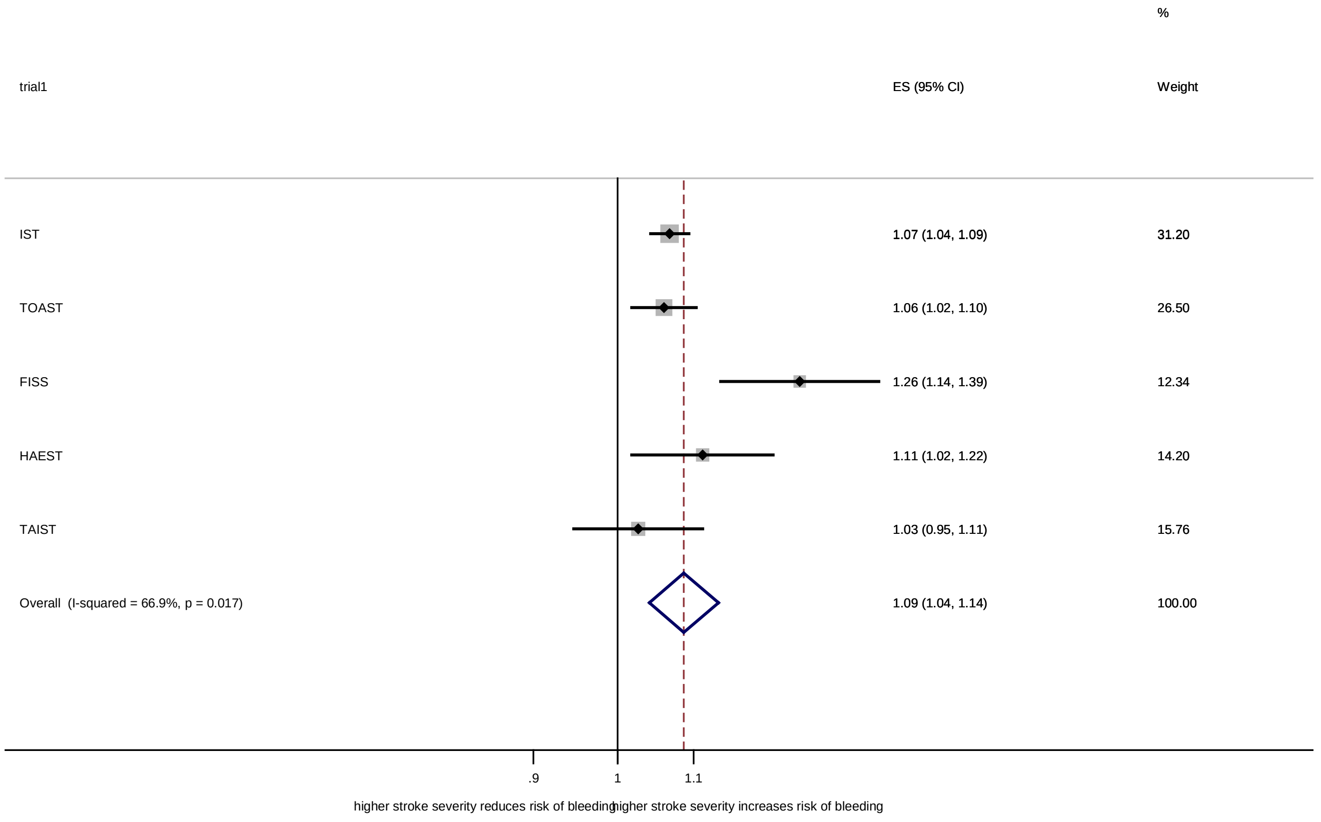

Supplementary figure 8. The odds ratio (ES) between a one year increase in NIHSS with haemorrhagic events 2 weeks post ischaemic stroke. IST: International Stroke Trial; TOAST: Trial of ORG 10172 in Acute Stroke Treatment; TAIST: Tinzaparin in Acute Ischaemic Stroke Trial; HAEST: Heparin in Acute Embolic Stroke Trial; FISS-tris: Fraxiparin in Stroke Study for the treatment of ischemic stroke. I squared: a measure of statistical heterogeneity.
